# The role of incline, performance level, and gender on the gross mechanical efficiency of roller ski skating

**DOI:** 10.3389/fphys.2013.00293

**Published:** 2013-10-22

**Authors:** Øyvind Sandbakk, Ann Magdalen Hegge, Gertjan Ettema

**Affiliations:** Center for Elite Sports Research, Department of Human Movement Science, Norwegian University of Science and TechnologyTrondheim, Norway

**Keywords:** men, metabolic rate, oxygen uptake, roller skiing, skating, women, work rate

## Abstract

The ability to efficiently utilize metabolic energy to produce work is a key factor for endurance performance. The present study investigated the effects of incline, performance level, and gender on the gross mechanical efficiency during roller ski skating. Thirty-one male and nineteen female elite cross-country skiers performed a 5-min submaximal session at approximately 75% of VO_2_peak on a 5% inclined treadmill using the G3 skating technique. Thereafter, a 5-min session on a 12% incline using the G2 skating technique was performed at a similar work rate. Gross efficiency was calculated as the external work rate against rolling friction and gravity divided by the metabolic rate using gas exchange. Performance level was determined by the amount of skating FIS points [the Federation of International Skiing (FIS) approved scoring system for ski racing] where fewer points indicate a higher performance level. Strong significant correlations between work rate and metabolic rate within both inclines and gender were revealed (*r* = −0.89 to 0.98 and *P* < 0.05 in all cases). Gross efficiency was higher at the steeper incline, both for men (17.1 ± 0.4 vs. 15.8 ± 0.5%, *P* < 0.05) and women (16.9 ± 0.5 vs. 15.7 ± 0.4%, *P* < 0.05), but without any gender differences being apparent. Significant correlations between gross efficiency and performance level were found for both inclines and genders (*r* = −0.65 to 0.81 and *P* < 0.05 in all cases). The current study demonstrated that cross-country skiers of both genders used less metabolic energy to perform the same amount of work at steeper inclines, and that the better ranked elite male and female skiers skied more efficiently.

## Introduction

The ability to efficiently utilize metabolic energy to produce work is a key factor for endurance performance (e.g., Bassett and Howley, 2000). It is well known that the metabolic cost of a muscle contraction increases linearly with the work rate produced during that contraction (Fenn, 1924), and a similar linear relationship has been found for whole body work against environmental resistance in endurance exercises (Cavanagh and Kram, 1985; Ettema and Loras, 2009). However, the intercept and slope of this relationship may differ between exercise modes due to various factors, such as technical complexity, continuous vs. discontinuous work rate generation, and body mass support.

The derivative of the relationship between work rate and metabolic rate, expressed as the mechanical efficiency, has been used for decades to express the metabolic effectiveness of the movement. In the present paper, we use gross efficiency, defined as the ratio of work generated to the total energy expended, to express the efficiency of the whole body (Cavanagh and Kram, 1985; Sidossis et al., 1992; Ettema and Loras, 2009). Experimental comparisons of gross efficiency may provide insight into how external conditions, technique, and performance level affect the total metabolic cost of a given work rate in endurance exercise.

In cycling, where the movement has low degrees of freedom and the possibilities to apply forces during a cycle remain the same across conditions, the work rate–metabolic rate relationship does not differ between world class and recreational cyclists nor across different inclines (Millet et al., 2002; Mognoni and di Prampero, 2003; Moseley et al., 2004). However, in more technically complex exercise modes, such as cross-country skiing where skiers adjust their technique with changing conditions, the work rate–metabolic rate relationship have been demonstrated to vary with changing incline and between world class and national class skiers (Sandbakk et al., 2010, 2012a). Specifically, a lower slope of the work rate–metabolic rate relationship and a greater gross efficiency have been found at steeper inclines (Sandbakk et al., 2012a) and recent studies show higher gross efficiency in better performing skiers (Sandbakk et al., 2010, 2011a,b).

Men and women perform at different work rates due to higher body masses and maximal capacities in men (Sandbakk et al., 2012b), but the work rate–metabolic rate relationship has been found similar in male and female elite skiers at moderate inclines (Sandbakk et al., 2012b; Ainegren et al., 2013). However, since female athletes are less examined than their male counterparts it still needs to be further investigated whether men and women have the same ability to convert metabolic energy into work rate at different terrain and if gross efficiency is influenced by incline and performance level to the same extent (Sandbakk et al., 2010, 2011a,b).

The purpose of the present study was to examine effects of incline, performance level, and gender on the gross mechanical efficiency during roller ski skating. We study elite skiers since they are experts in the movement and from a methodological standpoint provide us the opportunity to have highly standardized model subjects in this technically complex movement. Our hypotheses were that cross-country skiers of both genders would use less metabolic energy at steeper inclines when the work rate is kept constant and that better ranked skiers independent of gender would show higher gross efficiency.

## Methods

### Subjects

Thirty-one male and nineteen female cross-country skiers competing at national and international level volunteered to participate in the study. Their anthropometric and performance characteristics are documented in Table 1. All skiers were familiar with roller skiing on the treadmill from previous training and testing. The experimental procedures employed were approved by the Norwegian Regional Ethics Committee and the protocol and procedures explained verbally to each subject prior to obtaining written informed consent prior to participate.

**Table 1:** **Anthropometric and performance characteristics of 31 males and 19 females elite cross-country skiers (mean ± SD)**.

	**Men**	**Women**
Age (years)	22 ± 4	21 ± 3
Body mass (kg)	75 ± 5	61 ± 5
Body height (cm)	182 ± 3	169 ± 4
VO_2_peak(ml· min^−1^· kg^−1^)	70 ± 7	61 ± 6
Skating FIS points	43 ± 22	33 ± 14

### Instruments and materials

The roller ski skating was performed on a 6 × 3-m motor-driven treadmill (Bonte Technology, Zwolle, The Netherlands), the incline and speed were calibrated utilizing the Qualisys Pro Reflex system and Qualisys Track Manager software (Qualisys AB, Gothenburg, Sweden). The surface of the treadmill belt was covered with non-slip rubber and the subjects used their own poles (with a length = 91 ± 2% of body height) with special carbide tips and were secured with a safety harness during testing. To minimize variations in rolling resistance, all of the skiers used the same pair of Swenor roller skating skis with standard wheels (Swenor Roller skis, Troesken, Norway).

The rolling friction force (*F*_*f*_) of the roller skis was regularly determined by a towing test, described previously (Sandbakk et al., 2010), and the friction coefficient (μ) calculated by dividing *F*_*f*_ by the normal force (N), i.e., μ = *F*_*f*_ × *N*^−1^. There was less than 5% variation between the friction coefficient determined at different velocities and inclines, and between the different test days. The friction coefficient varied between 0.024 and 0.026 during the testing period, and the appropriate friction coefficient was used to calculate the work rate for each skier.

Ventilatory parameters were assessed continuously during all tests employing open-circuit indirect calorimetry with an Oxycon Pro apparatus (Jaeger GmbH, Hoechberg, Germany), as validated by Foss and Hallen (2005). Prior to each measurement, the VO_2_ and VCO_2_ analyzers were calibrated using a known mixture of gases (16.00 ± 0.04% O_2_ and 5.00 ±0.1% CO_2_, Riessner-Gase GmbH & Co, Lichtenfels, Germany) and the expiratory flow meter calibrated with a 3-L syringe (Hans Rudolph Inc., Kansas City, MO). Heart rate was recorded with a heart rate monitor (Polar RS800, Polar Electro OY, Kempele, Finland). In addition, the lactate concentration in whole blood (5-μL collected from the fingertip) was analyzed using the Lactate Pro LT-1710*t* kit (ArkRay Inc, Kyoto, Japan), which has been validated by Medbø et al. (2000).

Body mass was measured on a Kistler force plate (Kistler 9286AA, Kistler Instrument Corp., Winterthur, Switzerland) and body height with a calibrated stadiometer (Holtain Ltd., Crosswell, UK).

### Test protocols and measurements

Peak oxygen uptake was assessed using a standardized protocol as explained in detail elsewhere (Sandbakk et al., 2010); at a 5% incline using the G3 technique and with a starting speed of 16 km · h^−1^, the speed was increased by 1 km · h^−1^ every minute until exhaustion. This test was considered to represent maximal effort if VO_2_ reached a plateau despite increasing the speed and if the blood lactate concentration exceeded 8 mmol · L^−1^ (Bassett and Howley, 2000).

On two separate days in September or October, a 5-min submaximal session at 5 and 12% incline was performed after 20 min low-intensity warm-up and treadmill familiarization: The first session was performed at 75% of VO_2_peak using the G3 technique at the 5% incline, followed by G2 skating at the 12% incline (at a similar work rate as for 5%) on the second session. Pilot testing was performed with five representative subjects performing submaximal sessions at 75% of VO_2_peak using self-chosen, G2 and G3 techniques at both inclines, and showed that the sub-techniques used in this study were the skiers' self-chosen and most efficient ones at the representative incline. The G3 skating technique (also referred to as V2, 2-skate, and double dance) is typically used at slight to moderate uphills and involves a symmetrical double poling action together with every leg push-off. The G2 skating technique (also referred to as offset and V1 skate) is used at steep uphills and involves an asymmetrical double poling action together with every second leg push-off.

The metabolic rate was calculated from VO_2_ and VCO_2_ in aerobic steady state conditions, obtained during the last minute of each test, as the product of VO_2_ and the oxygen energetic equivalent using the associated respiratory exchange ratio and standard conversion tables (Peronnet and Massicotte, 1991). The work rate was calculated as the sum of power against gravity [*P*_*g*_ = *m* · *g* · sin (α) · *v*] and friction [*P*_*f*_ = *m* · *g* · cos (α) · μ · *v*]; where *m* is the mass of the skier, *g* the gravitational acceleration 9.81 m s^−2^, α the angle of treadmill incline, *v* the belt speed and μ the frictional coefficient. Gross efficiency was calculated as the external work rate divided by the metabolic rate, in accordance with Sandbakk et al. (2010). Gross efficiency is dependent on the absolute level of work rate, caused by the non-zero y-intercept of the work rate–metabolic cost relationship (Ettema and Loras, 2009), meaning that gross efficiency is substantially lower with low than with high work rates (e.g., at lower performance levels). Therefore, we also tested the relationship between gross efficiency and performance level when gross efficiency was corrected for differences in work rate.

### Performance level

The individual level of skiing performance on snow was assessed based on the Federation of International Skiing (FIS) points collected during the 4 months directly following treadmill testing. According to FIS, a skier's rank is established relative to a zero-point standard by the top-ranked skier in the world, meaning that better skiers have fewer FIS points. A skier's total score for a given race is determined by adding race points (from comparing the individual skier's time with the winner's time) and race penalty. Here, we used the average of the three best FIS points in individual skating distance races.

### Statistical analysis

All data were checked for normality and presented as mean and standard deviation (SD). Comparisons of physiological, mechanical, and performance variables between genders were analyzed using the independent *t*-test procedure, whereas pair-wise differences for physiological and mechanical variables between the two inclines were identified by a paired samples *t*-test. Linear regression and correlations between the same variables within the various conditions were tested for all skiers together and within groups (both inclines and genders) using Pearson's product-moment correlation coefficient test. Repeated measurement of the physiological parameters when roller skiing at a constant submaximal speed on the treadmill demonstrated intraclass correlation coefficients > 0.95. Statistical significance was set at *P* < 0.05. All statistical tests were processed using SPSS 11.0 Software for Windows (SPSS Inc., Chicago, IL).

## Results

The average testing speeds for men and women at 75% of VO_2_peak were 13.7 ± 1.4 and 12.4 ± 1.2 km · h^−1^ at the 5% incline and 7.3 ± 0.8 and 6.6 ± 0.6 km · h^−1^ at the 12% incline. The corresponding physiological values for men and women were oxygen uptakes of 3.66 ± 0.39 and 2.81 ± 0.26 L · min^−1^ at the 5% incline and 3.32 ± 0.34 and 2.71 ± 0.29 L · min^−1^ at the 12% incline, respiratory exchange values of 0.90 ± 0.03 and 0.90 ± 0.02 at the 5% incline and 0.91 ± 0.03 and 0.90 ± 0.03 at the 12% incline and blood lactate concentrations of 1.6 ± 0.4 and 1.4 ± 0.3 mmol · L^−1^ at the 5% incline and 1.5 ± 0.4 and 1.5 ± 0.4 mmol · L^−1^ at the 12% incline.

**Figure 1:**
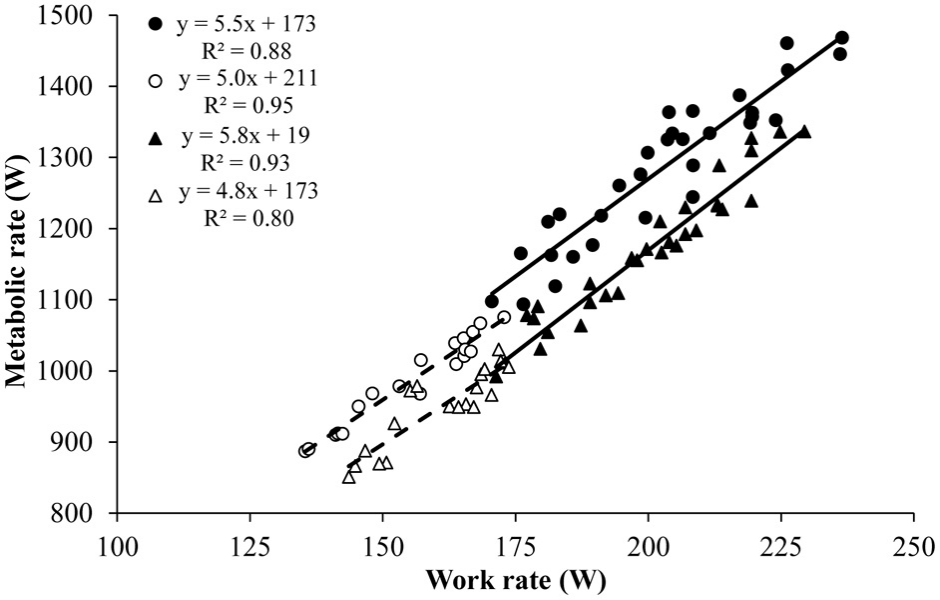
**Work rate–metabolic rate relationship for 31 males and 19 females elite cross-country skiers while roller ski skating on a 5 and 12% inclined treadmill in the G3 and G2 skating technique, respectively**. Individual values and trend lines (based on linear regression) are shown for men (filled symbols, solid line) and women (open symbols, dashed line) and for the 5% (circular symbols) and 12% incline (triangular symbols). The correlation coefficients between gross efficiency at the two inclines were 0.55 and 0.72 for men and women respectively (both *P* < 0.05).

A linear relationship between work rate and metabolic rate was demonstrated for both men and women at the 5 and 12% incline (Figure 1; all *P* < 0.05). The regression slope of the work rate–metabolic rate relationship did not differ between the two inclines nor between gender, but the regression line for the 5% incline was significantly elevated compared to the 12% incline, due to a higher intercept, for both genders (both *P* < 0.05). The intercept of the work rate–metabolic rate relationship was significantly higher for men than for women at the 5% incline (*P* < 0.05).

**Figure 2:**
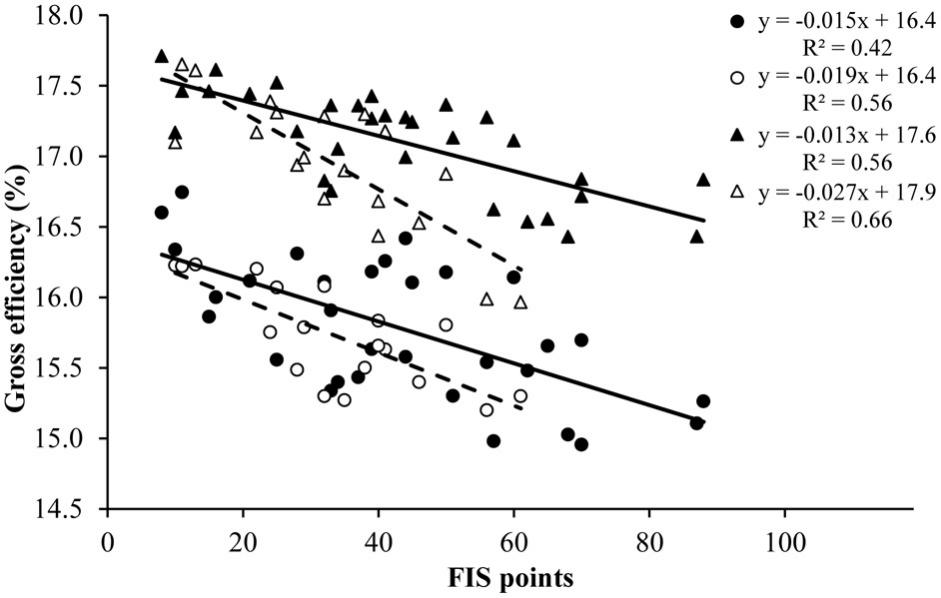
**Gross efficiency plotted against FIS points for 31 males and 19 females elite cross-country skiers while roller ski skating on a 5 and 12% inclined treadmill in the G3 and G2 skating technique respectively**. Individual values and trend lines (based on linear regression) are shown for men (filled symbols, solid line) and women (open symbols, dashed line) and for the 5% (circular symbols) and 12% incline (triangular symbols).

Significant correlations between gross efficiency and FIS points (as a measure of performance level) were found for men and women at both inclines (Table 2 and Figure 2; all *P* < 0.05). The correlation coefficients between work rate and FIS points were −0.38 for men at both inclines, and −0.44 and −0.41 for women at the 5 and 12% incline respectively (all *P* < 0.05). Therefore, the effects of work rate on the relationship between FIS-points and gross efficiency had to be accounted for. This was done in two steps: Initially, the measured metabolic rate was subtracted from the estimated value for metabolic rate at each individual's work rate (calculated with linear interpolation from the work rate–metabolic rate regression line), so that a lower value represents a lower gross efficiency. Thereafter, the outcomes were correlated against FIS-points, which gave slightly reduced, but still significant correlations (Table 2; all *P* < 0.05). There was a tendency (*P* = 0.058) toward lower FIS points in women compared to men. This difference was mainly caused by two male skiers with lowest FIS points (see Figure 2), and the analyses without these subjects revealed that all main findings remained the same.

**Table 2:** **FIS points correlated against gross efficiency (GE) and work rate accounted efficiency for 31 male and 19 female elite cross-country skiers while roller ski skating on a 5 and 12% inclined treadmill in the G3 and G2 skating technique respectively**.

**Incline**	**Group**	**GE**	**E-WR**
5%	Men	*r* = −0.65^*^	*r* = −0.55^*^
	Women	*r* = −0.75^*^	*r* = −0.63^*^
	All pooled	*r* = −0.64^*^	*r* = −0.53^*^
12%	Men	*r* = −0.75^*^	*r* = −0.72^*^
	Women	*r* = −0.81^*^	*r* = −0.72^*^
	All pooled	*r* = −0.65^*^	*r* = −0.69^*^

E-WR, the measured metabolic rate was subtracted from the estimated value for metabolic rate at each individual's measured work rate (using linear interpolation of the work rate–metabolic rate relationship).

* Significant correlation coefficient, P < 0.05.

Gross efficiency increased linearly with increasing work rate for both men and women at the 5% incline (all *P* < 0.05). Men worked at higher work rates (and metabolic rates) than women (all *P* < 0.05), but without any significant differences in gross efficiency between genders (Table 3). At the matched work rates, the metabolic rates were lower and gross efficiency higher at the 12% incline compared to 5% for both gender groups (Table 3; *P* < 0.05).

**Table 3:** **Work rate, metabolic rate and gross efficiency in 31 males and 19 females elite cross-country skiers while roller ski skating on a 5 and 12% inclined treadmill in the G3 and G2 skating technique respectively at similar work rates (mean ± SD)**.

**Variables**	**Gender**	**Incline**	
		**5%**	**12%**
Work rate (W)	Men	203 ± 18^#^	200 ± 16^#^
	Women	156 ± 12	161 ± 10
	All pooled	185 ± 28	185 ± 24
Metabolic rate (W)	Men	1286 ± 106^#^	1168 ± 96^*^^#^
	Women	987 ± 62	948 ± 55^*^
	All pooled	1172 ± 173	1084 ± 135^*^
Gross efficiency (%)	Men	15.8 ± 0.5	17.1 ± 0.4^*^
	Women	15.7 ± 0.4	16.9 ± 0.5^*^
	All pooled	15.8 ± 0.4	17.0 ± 0.4^*^

* Significantly different from the corresponding value at the 5% incline, P < 0.05.

# Significantly different from the corresponding value for women, P < 0.05.

## Discussion

The current study examined the effects of incline, performance level, and gender on the gross efficiency of roller ski skating in elite cross-country skiers. The main findings were that cross-country skiers of both genders used less metabolic energy to perform the same amount of work at steeper inclines and that better ranked skiers showed higher gross efficiency. Furthermore, there were no differences in gross efficiency between male and female elite skiers.

In the current study elite skiers consistently attained lower metabolic rates on the 12% than on the 5% incline when roller skiing at similar work rates. This confirms previous studies where a lower regression line of the work rate–metabolic rate relationship, and thereby a higher gross efficiency, has been found at steeper inclines (Sandbakk et al., 2012a). One explanation for this might be the more continuous force generation and shorter passive phases (i.e., gliding and swing times) with the lower speeds executed at steeper inclines (Sandbakk et al., 2012a). In a previous study the same technique was executed at different inclines (Sandbakk et al., 2012a), whereas the current study examined the most efficient technique at the representative incline. However, both studies clearly show that the work rate–metabolic rate relationship is influenced by incline.

The higher efficiency at the steeper incline may partly explain why skiers use higher work rates on uphill terrain than on the flat during competitions on varying terrain (Norman and Komi, 1987; Norman et al., 1989; Sandbakk et al., 2012a). This is different from cycling where incline has a negligible effect on the ability to produce work (Millet et al., 2002). This difference between cycling and skiing probably reflects the difference in technique between these exercise modes, caused by more discontinuous movements and higher degrees of freedom when coordinating the upper and lower extremities during skiing. Thus, skiing efficiency is lower than in cycling and more influenced by external conditions.

The current study shows that better ranked skiers of both genders have higher gross efficiency, which extends previous findings that have highlighted gross efficiency as an important determinant of skiing performance level (Sandbakk et al., 2010, 2011a,b). Notably the highest correlation coefficient between FIS points and gross efficiency was at the steepest incline, which supports previous research that uphill inclines are the most performance determining parts of a race (Norman and Komi, 1987; Norman et al., 1989; Sandbakk et al., 2012a). Previously, Ainegren et al. (2013) showed that elite skiers of both genders had higher gross efficiency than recreational skiers. However, the role of efficiency on performance level within elite skiers was examined for the first time among women in the current study, and shows that women's performance level is associated with efficiency to the same extent as men. Overall, the current findings further strengthen the importance of a high efficiency for endurance performance among male and female cross-country skiers.

In contrast to the 13% difference in maximal capacity between men and women, which is comparable to a previous study by our group (Sandbakk et al., 2012b), the work rate–metabolic rate relationship did not differ substantially between men and women in ski skating.

Even though differences in work rate had a significant influence on gross efficiency, there was no significant gender difference in the gross efficiency at either incline. The regression lines for men and women were almost identical at the 12% incline, while at the 5% incline the regression line for men was slightly elevated, caused by a higher intercept, compared to the corresponding one for women. This latter difference was not found in a previous study where both slopes and intercepts were close to identical for male and female skiers at a 5% incline (Sandbakk et al., 2012a). The reason for this is unknown, but might be sought in technique differences and/or the small, but non-significant, higher performance level in women. While the slightly elevated values for men may be of interest, the apparent higher y-intercept (i.e., metabolic rate of unloaded work), seems less meaningful since this intercept is only is a mathematically extrapolation value. If the value has physiological meaning, it must first be established that the linear relationships holds for the entire work rate range from zero watt and upwards. This is unlikely since moving without any external resistance in a coordinated manner is challenging and likely requiring an excessive amount of energy that is not accounted for at moderate and higher work rates. Nevertheless, the current study together with our previous paper on gender differences (Sandbakk et al., 2012b) suggest that male and female skiers do not differ substantially in the ability to convert metabolic rate into work rate both on relatively flat and uphill terrain.

## Conclusions

The current study shows that cross-country skiers of both genders use less metabolic energy to perform the same amount of work at steeper inclines. Better ranked skiers of both genders show higher gross efficiency which confirms that gross efficiency has an important role for performance in cross-country skiing. However, male and female elite skiers do not differ in gross efficiency, indicating that male and female skiers substantially possess the same ability to convert metabolic energy into work rate and speed.

### Conflict of interest statement

The authors declare that the research was conducted in the absence of any commercial or financial relationships that could be construed as a potential conflict of interest.
